# Strategically influencing an uncertain future

**DOI:** 10.1038/s41598-020-69006-x

**Published:** 2020-07-22

**Authors:** Alain Govaert, Ming Cao

**Affiliations:** Engineering and Technology Institute Groningen (ENTEG), Faculty of Science and Engineering, University of Groningen, Groningen, 9747 AG The Netherlands

**Keywords:** Applied mathematics, Human behaviour, Cooperation

## Abstract

Many of today’s most pressing societal concerns require decisions which take into account a distant and uncertain future. Recent developments in strategic decision-making suggest that individuals, or a small group of individuals, can unilaterally influence the collective outcome of such complex social dilemmas. However, these results do not account for the extent to which decisions are moderated by uncertainty in the probability or timing of future outcomes that characterise the valuation of a (distant) uncertain future. Here we develop a general framework that captures interactions among uncertainty, the resulting time-inconsistent discounting, and their consequences for decision-making processes. In deterministic limits, existing theories can be recovered. More importantly, new insights are obtained into the possibilities for strategic influence when the valuation of the future is uncertain. We show that in order to unilaterally promote and sustain cooperation in social dilemmas, decisions of generous and extortionate strategies should be adjusted to the level of uncertainty. In particular, generous payoff relations cannot be enforced during periods of greater risk (which we term the “generosity gap”), unless the strategic enforcer orients their strategy towards a more distant future by consistently choosing “selfless” cooperative decisions; likewise, the possibilities for extortion are directly limited by the level of uncertainty. Our results have implications for policies that aim to solve societal concerns with consequences for a distant future and provides a theoretical starting point for investigating how collaborative decision-making can help solve long-standing societal dilemmas.

## Introduction

If individuals choose between rewards that differ only in amount, timing, or certainty, decisions are relatively predictable because general principles of choice apply^1^. For example, individuals tend to choose higher rewards over lower ones, sooner rewards over later ones, and secure rewards over risky ones. Indeed, such decisions make sense from both an economic and evolutionary perspective and are observed in both humans and animals^1,2^. Predicting decisions becomes more challenging when the choice options differ in a combination of these factors. For example, it can be difficult to predict how an individual chooses between a small but immediate reward and a large but distant one. Although such combinations of different features usually require trade-offs in decision-making, their salient features can be studied from the perspective of *discounting* on the basis of the expected time (delay discounting) or likelihood of their occurrence (probability discounting)^1,3–5^. Indeed, these discounting methods are positively correlated^4,5^.

Game theory provides a unifying framework through which these decision-making processes can be formalised under a variety of complex situations. The defining feature of *games* is that their outcomes depend on not only one’s own decision, but also on the decisions of others. This interdependence inherently causes uncertainty in *probable* outcomes and payoffs. It becomes more challenging in *repeated* games in which a series of interactions occur over time and individuals need to make strategic decisions that take into account how their past and current decisions can influence *future* payoffs under reciprocal altruism, antagonism, punishment or reward^6–8^.

Of particular interest are social dilemmas in which immediate self-interests conflict with long-term collective interests. In these complex social and economic situations, discounting and reciprocity have been shown to interactively influence the level of “selfless” cooperative decisions^9–11^. This interaction also serves as a plausible explanation for the changing (cooperative) behaviours throughout the life span of humans^12–14^.

Understanding if, and how, strategic decisions are affected by delay and probability discounting is becoming more and more important because many of today’s most pressing societal concerns, like climate change, require current decisions to take into account the consequences for an *uncertain* distant future^15–17^. In these complex settings, theoretical models for both delay and probability discounting methods use discount rates that decrease over time^2,18–22^. These hyperbolic-like functions have indeed proven to be a better fit to empirical discounting rates than traditional time-consistent exponential functions that tend to discount the far future too fast^1,21,23^.

Recent developments in the theory of direct reciprocity and strategic behaviour suggest that a single, or small group of strategic individuals, can have a much larger influence on other players’ decisions than previously anticipated^24,25^. In particular, these theories enable strategic individuals to solve social dilemmas by applying generous strategies that can unilaterally “enforce” mutual cooperation in a large group of decision-makers^25,26^. However, these theories are built on traditional time-consistent discounting methods, that leave out important elements of the psychology of discounting^16^. In fact, the current assumptions on discount factors in repeated social dilemmas can easily cause discrepancies between theoretical cooperation levels and observed experimental behaviours^27,28^.

Although these novel theories provide important perspectives for policy-makers when exerting influence in long-run collective outcomes, it is not yet known how the intricate strategies hold up under more sophisticated discounting methods that take the inherent uncertainty of future outcomes into account. By incorporating uncertainty about the discount factor into the framework of repeated games, we generalise the existing theories on strategic play and show how individuals can exert a significant level of influence even under time-inconsistent discounting. The proposed discounting framework is consistent with the hyperbolic form observed by experimentalists and, in its deterministic limits, complies with existing theories of strategic play. We postulate that this theoretical framework is more appropriate for describing real-world decision making procedures in which judgements on the number of interactions is made under uncertainty^27^ or the far-distant future is crucial for the success of current strategic decisions^16^.

**Figure 1:**
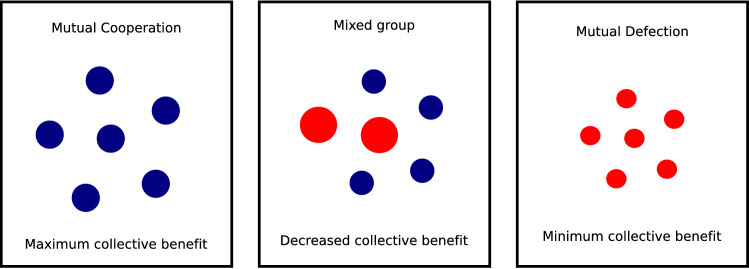
Illustration of a social dilemma. Dots represent players: cooperators are indicated by blue dots and defectors by red dots. The size of the dot indicates the payoff of the player. A group of cooperators typically create a maximum collective benefit. In mixed groups defectors benefit from cooperators and receive larger payoffs. When all individuals defect the collective benefit is typically minimum as in the “tragedy of the commons”^29^.

To show the utility of our results, we consider a general class of *n*-player social dilemmas^15,16,25^. In the model individual *players* repeatedly choose to cooperate or defect. A player’s payoff in a given round depends on their decision and the number of cooperating co-players^25,30,31^. If $z\in \{0,1,\dots ,n-1\}$ co-players cooperate, then the single-round payoff for cooperation is $a_z$, and the single-round payoff for defection is $b_z$. We only assume the single-round payoffs satisfy three characteristic properties of social dilemmas^25,32^: first, irrespective of one’s own decision to cooperate or defect, players prefer their co-players to cooperate; second, in a group of cooperators and defectors, defecting players have a strict advantage; finally, the mutual cooperation payoff ($a_{n-1}$) is more beneficial than the mutual defection payoff ($b_0$), see  Fig. 1. These characteristics are able to capture a variety of complex situations in which payoffs can non-linearly depend on the decisions of one’s co-players and include the prisoners dilemma game, the public goods game, the volunteers dilemma, the *n*-player snowdrift game^31^, the *n*-player stag hunt game^33^, and many more.

### Discounting an uncertain future

In traditional repeated games with finite but undetermined time horizons, the expected number of rounds is determined by a fixed and common *discount factor* $\delta \in (0,1)$ that, given the current round of interactions, determines the probability of a next round, and is therefore also referred to as a *continuation probability*. Consequently, *expected* discounted payoffs are calculated using a discounting function $\delta ^t$ that corresponds to deterministic discrete-time exponential discounting^16,34,35^. However, if one is uncertain about the discount factor or the probability for next interactions^27^, then the value of the payoffs relying on future interactions are uncertain as well and it is not the case that a fixed parameter $\delta$ can be used to represent the discounted value of payoffs.

**Figure 2:**
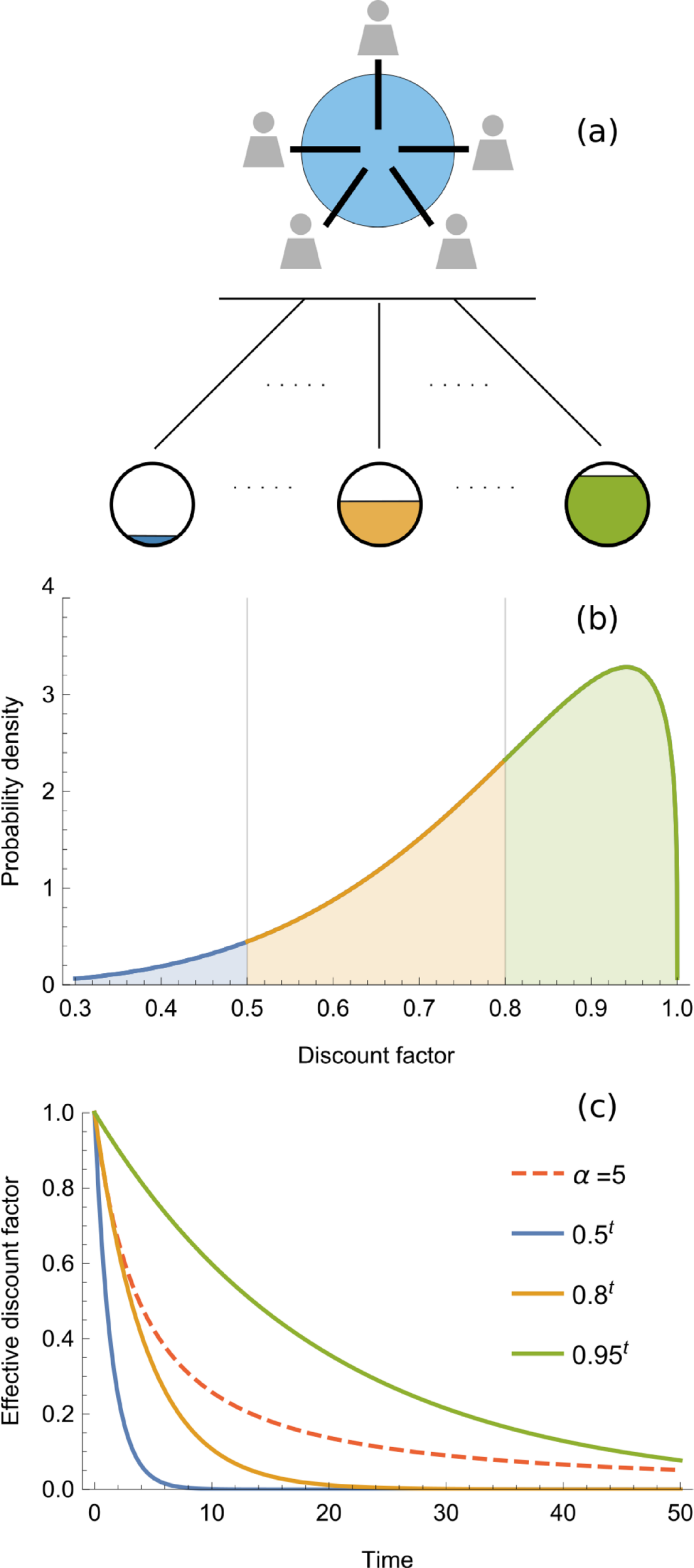
Discounting uncertain future outcomes. Strategic decisions in social dilemmas are affected by the valuation of probabilistic long-run outcomes that result from collective behaviour and the probability for continued interaction. Different outcomes, indicated by the partially filled coloured circles in panel (**a**), can be valued with different discount factors depending on expected time and likelihood of occurrence. Theoretically these psychological and economic complexities in decision-making can be modelled by an uncertain discount factor shown in panel (**b**). This results in the hyperbolic-like effective discounting function shown in panel (**c**) and equation (1). In the illustration the shape parameters of the beta distribution in panel (**b**) are $\alpha =5$ and $\beta =\frac{5}{4}$, leading to a mean discount factor of 0.8 and a variance of approximately 0.022.

Under this uncertainty, hyperbolic discounting functions typically refer to relatively short-run decision-making behaviour under delayed or probabilistic rewards^5,23,36,37^. A similar argument can be made for discounting the distant future: strategic decisions with consequences for the distant future are made not knowing the relevant outcome and should therefore be discounted probabilistically^21,23^. In the spirit of *gamma discounting*^21^, let us thus assume that discount factors are described by a *random variable* *x*, whose probability density function $f(x,\alpha ,\beta )$, defined for all $x\in [0,1]$, is of the *beta form*

$$\begin{aligned} f(x,\alpha ,\beta ):=\frac{x^{(\alpha -1)}(1-x)^{(\beta -1)}}{\mathrm {B}(\alpha ,\beta )}, \quad \alpha ,\beta \in \mathbb {R}_{+}, \end{aligned}$$

where $\mathrm {B}(\alpha ,\beta )$ is the beta function. Indeed, the beta distribution is often used to describe the distribution of a bounded random variable (like an uncertain probability) and is thus a suitable choice^38,39^. The obtained *effective discounting function*^21^ becomes

1 $$\begin{aligned} d(t):=\int _0^1 x^t f(x,\alpha ,\beta )dx=\frac{\Gamma (t+\alpha )\Gamma (\alpha +\beta )}{\Gamma (t+\alpha +\beta )\Gamma (\alpha )}, \end{aligned}$$

where $\Gamma (\cdot )$ indicates the gamma function. This effective discounting function indicates how payoffs are discounted when the probability for future interactions and their outcomes are uncertain (Fig. 2).

As one would expect, the payoffs that are received *now* are not subject to uncertainty and are “discounted” by the factor $d(0)=1$. Interestingly, the *rate of change* of equation (1) is

2 $$\begin{aligned} -\frac{\beta }{t+\alpha +\beta },\quad t\ge 0, \end{aligned}$$

and thus supports the empirically validated feature of hyperbolic discounting in which the discount rate *decreases* monotonically over time^1, 2^ and thereby suitably discounts the distant future with the lowest possible rate^21,23^.

To theoretically investigate how this affects *strategic decision-making*, one can incorporate the effective discount function in equation (1) in a *repeated game*. Denoting by $\pi _i(t)$ the expected payoff of player *i* in round *t*, the *average discounted payoff* of player *i* can be written as

3 $$\begin{aligned} \pi _i:=\frac{\sum _{t=0}^\infty d(t)\pi _i(t)}{\sum _{t=0}^\infty d(t)}. \end{aligned}$$

For $\beta >1$ the series of the effective discounting function in the denominator of equation (3) *converges* to

4 $$\begin{aligned} \frac{\alpha +\beta -1}{\beta -1}, \end{aligned}$$

indicating that the shape parameters of the beta distribution analytically determine the normalisation factor of the average discounted payoff of players. It is worth pointing out that the requirement $\beta >1$ rules out the possibility for a uniform and u-shaped distribution, indicating that players cannot be “completely” uncertain about how to value an uncertain future.

### Influencing an uncertain future

A strategic individual is typically interested in maximising their influence in a decision-making process by employing a decision-making strategy that guarantees a desired relative performance. One could, for instance, be interested in outperforming others via *extortionate* ZD strategies or ensuring that others do well via *generous* ZD strategies^24,25,40^. When there is no discounting or future payoffs are discounted *deterministically*, individuals can indeed strategically influence outcomes by employing a *fixed* strategy that enforces a linear payoff relation in the average discounted payoff of their co-players ($\pi _{-i}$) and their own average discounted payoff^24,25,35^:

5 $$\begin{aligned} \pi _{-i}=s\pi _i+(1-s)l. \end{aligned}$$

The strategy parameter *s* is commonly referred to as the *slope* of the linear payoff relation and determines how $\pi _{-i}$ varies with $\pi _i$, while the parameter *l* is referred to as the *baseline payoff* that determines the average discounted payoffs when all players employ the same ZD strategy^25^. While a fixed strategy suffices in the deterministic case, equation (2) indicates that uncertainty does change discount rates, which have to be taken into account in one’s effort to strategically influence uncertain future outcomes. This necessarily requires one’s decision-making strategy to adapt to the changing discount rates and thus become *time-varying*. In section 2 of the Supplementary Information we show how *risk-adjusted strategies* cope with uncertainty and by doing so, allow a strategic player to strategically influence an uncertain future. However, the uncertainties, that are so common in the real-world, come with fundamental limitations that previous theories have overlooked.

### The generosity gap

**Figure 3:**
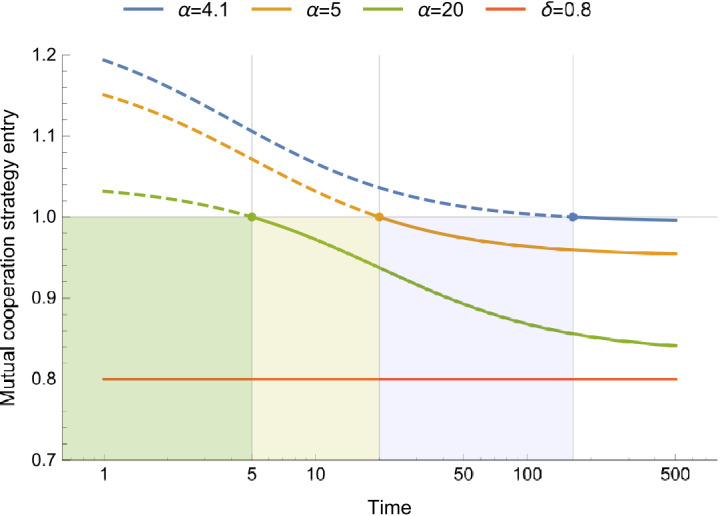
The generosity gap. Numerical example of the conditional probability for the strategic player to mutually cooperate with their co-players for a variable shape parameter $\alpha$, a fixed mean discount factor $\mu =0.8$, and $\beta =\frac{\alpha (1-\mu )}{\mu }$. When the variance in the discount factor increases (for lower $\alpha$ values) it takes longer for the generous risk-adjusted strategy to become well-defined. Consequently, the generosity gap, that indicates the time the strategic player cannot enforce a generous payoff relation, increases in length.

Strategies that can enforce generous payoff relations ($0<s<1,l=a_{n-1}$) have received significant scientific attention for their ability to unilaterally promote and sustain cooperative behaviour via direct reciprocity^7,25,26^ and evolution^40,41^. We find that such strategies *do not exist* when the uncertain future is discounted using the hyperbolic-like effective discounting function in equation (1) (see Supplementary Information section 2 for more details). Due to the time-varying discount rates these strategies become well-defined only after a significant amount of time, i.e. the *generosity gap* (see Fig. 3), has passed:

6 $$\begin{aligned} t\ge \frac{\alpha }{\beta -1}, \quad \beta >1. \end{aligned}$$

Equation (6) implies that the more uncertain an “as if” constant mean discount factor becomes, the longer a strategic player is prevented from enforcing a generous payoff relation, unless they simply *always* cooperate (see Supplementary Information section 2 for details). After the generosity gap has passed, the effective discounting function has decreased to such an extent that a desired generous payoff relation can be enforced, but *only* over the averaged payoffs received beyond the generosity gap that are discounted with a relatively *constant* and low discount rate. This indicates that under uncertainty a generous strategic player can only solve social dilemmas by completely setting aside their immediate and short-term interests and adjust their strategic influence to a notably far-distant future. Interestingly, if the discount factor becomes certain, the deterministic limits of equation (1) are consistent with existing theories in which generous payoff relations can be enforced without any generosity gap (see the Supplementary Information section 2 for more details).

### Extortion in an uncertain future

When future interactions are at least as likely as a termination of the game, the beta distribution is symmetric or negatively skewed ($\alpha \ge \beta$) and strategic decisions tend to include at least one future interaction. In the Supplementary information (section 2) we show that for many social dilemmas, in fact, this is a requirement for the possibility to strategically influence an uncertain future with an *extortionate* payoff relation ($0<s<1, l=b_0$) that can promote cooperation and typically ensures a beneficial relative performance of the strategic player. For any positively skewed distribution the low mean discount factor does not allow strategic influence because payoffs are discounted too fast and others cannot “learn” to cooperate with the extortioner^7,26^. This additional requirement also provides insight into *how* uncertain the discount factor or continuation probability can be before losing the possibility to enforce a desired extortionate payoff relation. For symmetric or negatively skewed distributions the theoretical *maximum variance* that a strategic player can deal with while exerting an extortionate payoff relation occurs when $\alpha =\beta$, and evaluates as

7 $$\begin{aligned} \sigma _{\text {max}}^2=\frac{1}{4(2\alpha +1)}<\frac{1}{12}. \end{aligned}$$

**Figure 4:**
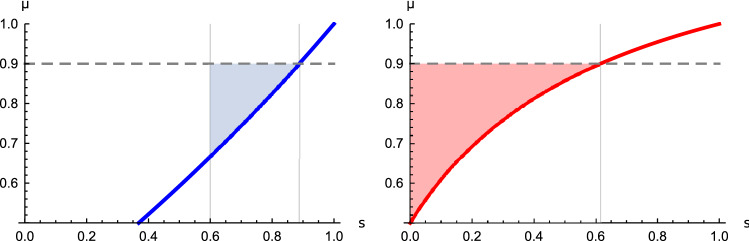
Enforceable slopes and mean discount factors. Left: Numerical examples of enforceable slopes by extortionate strategies when $\mu =\frac{9}{10}$. In an *n*-player snowdrift game with benefit $b=\frac{5}{4}$, cost $c=1$ and $n=3$, extortionate strategies can only enforce slopes after the vertical line at $s=1-\frac{c}{b(n-1)}=\frac{6}{10}$. Every slope *s* for which the blue curve is below $\mu$ is enforceable (blue region). Right: in a linear public goods game with multiplier $r=\frac{12}{10}$, cost $c=1$ and $n=3$, extortionate strategies can enforce any slope *s* for which the red curve is under $\mu$, this is indicated by the red region. See the Supplementary Information section 3 for detailed descriptions of the games.

Now let us suppose the strategic player has estimated the distribution of the discount factor^21^. Then, exactly how extortionate can a payoff relation be? In general, this depends on the one-shot payoffs and the mean of the beta distribution given by $\mu =\frac{\alpha }{\alpha +\beta }$. Figure 4 illustrates this for the linear public goods game and the *n*-player snowdrift game^31^. In both games, an increased mean discount factor slows down discounting and enables more extortionate influence (see Supplementary Information section 2 for a general characterisation). However, as with generosity there is a catch: an increased mean discount factor comes at the price of a *decreased* maximum allowable variance as determined by equation (7). Thus, when discounting becomes *slower* and the distant future becomes more relevant for today’s decisions, an extortioner is required to be more certain about the valuation of events further in the future.

## Discussion

Classic theories of strategic decision-making rely on how one’s actions can affect their future. If one would consider to defect by choosing selfishly at some point in time, how large will the consequences of retaliation be? And is the fear of retaliation from others enough to sustain cooperation even when the immediate benefit of defection is large? These strategic trade-offs are commonly referred to as “the shadow of the future” and provide an elegant theoretical explanation for the emergence of cooperative behaviour of rational players in repeated social dilemmas^8^. However, even with moderate discount factors, the exponential discounting functions used in these theories attribute meaningless significance to the distant future^23^ and do not take into account empirically observed time-inconsistent valuations, making them less suitable for modelling strategic decisions that affect a distant future. More recently, strategic behaviour has been studied from an alternative perspective by identifying decision-making strategies that can unilaterally exert strategic influence on the long-run collective behaviour. Because they require minimal assumptions on the behaviour of others, such strategies are of particularly interest to human decision-making^7^. However, also these theories are built upon valuations of future scenarios that, in reality, are riddled with uncertainties in the probability or timing of payoffs that are likely to influence strategic decisions^27,42^.

Here we have modelled these uncertainties with a discounting method that exhibits the characteristic features of empirically validated delay, probability and social discounting methods^1,5^. Using the proposed framework, existing theories of strategic decision-making can be recovered in deterministic limits and new insights are obtained into the interaction between uncertainty, discounting and the possibilities for strategic influence. Namely, in social dilemmas, uncertainty leads to generosity gaps that require generous strategic influence to be adjusted to the longer term. These potentially long periods of time in which no generous payoff relation can be enforced may also contribute to the empirically observed inconsistencies in strategic influence and cooperation levels over time^25,26^. On the other hand, our results indicate that the slower discounting becomes, the more certain an extortioner needs to be about an increasingly distant future: sufficient patience thus requires sufficient certainty. These findings illustrate the difficulties one can expect when attempting to exert strategic influence in the real world and provide new insights for decision-making experiments in more controlled environments. From a more technical point of view, our extension to time-varying strategies that is found in the Supplementary Information section 2, provides a novel perspective for the study of reciprocity in *changing* environments^43^.

In this paper, we interpreted the beta distribution as a *common* uncertain belief in the discount factor or continuation probability which is a rather restricting assumption. However, we believe arguments can be made for interpreting the beta distribution as an approximation of the distribution of discount factors in a large group of individuals^21,44^. In this case, (1) can be seen as a weighted average discounting function used in *collaborative decisions*^45,46^. In this context, our framework can be used to theoretically study the strategic behaviour of groups making collective decisions and how the group composition can affect their cooperative behaviour.

Regardless of the interpretation, our work shows that strategic efforts to solve social dilemmas must be adjusted to the uncertainty in the valuation of the future, because only then can strategic influence help to solve today’s societal concerns.

## Supplementary Information

Supplementary Information.
