# Supplementary material for: Strategically influencing an uncertain future
Source: Sci Rep. 2020 Jul 22;10:12169. doi: 10.1038/s41598-020-69006-x (PMC7376051; doi:10.1038/s41598-020-69006-x)
Supplement: Supplementary file 1 — Supplementary Information. [file 41598_2020_69006_MOESM1_ESM.pdf]

Supplementary Information for "Strategically influencing an uncertain future"

Alain Govaert, Ming Cao

July 9, 2020

Contents

1 Beta discounting in repeated games **1**

2 Risk-adjusted strategies in repeated games **2**

2.1 The existence of risk-adjusted strategies 5

2.1.1 The generosity gap 6

2.1.2 Characterizing enforceable payoff relations of risk-adjusted strategies 6

2.1.3 Uncertainty and the level of extortion 10

3 Definitions of n-player games in main text **11**

3.1 The n-player snowdrift game 11

3.2 The n-player linear public goods game 12

4 References **12**

1 Beta discounting in repeated games

We start with describing in detail the probabilistic discounting framework presented in the main text. Let us assume the discount factors are described by a *random variable* x, whose probability density function $f(x, \alpha, \beta)$, defined for all $x \in [0, 1]$, is of the beta form

$$f(x, \alpha, \beta) := \frac{x^{(\alpha-1)}(1-x)^{(\beta-1)}}{\mathrm{B}(\alpha, \beta)}, \tag{S1}$$

where $\mathrm{B}(\alpha, \beta)$ is the Beta function:

$$\mathrm{B}(\alpha, \beta) = \frac{\Gamma(\alpha)\Gamma(\beta)}{\Gamma(\alpha+\beta)}.$$

The mean and variance of the beta distribution are, as usual, defined as

$$\mu = \frac{\alpha}{\alpha+\beta}, \quad \sigma_x^2 = \frac{\alpha\beta}{(\alpha+\beta)^2(\alpha+\beta+1)}.$$

The effective discount function is

$$d(t) := \int_0^1 x^t f(x, \alpha, \beta) dx, \tag{S2}$$

which for $t \geq 0$, $\beta > 1$ and $\alpha > 0$ evaluates as

$$d(t) = \frac{\Gamma(t+\alpha)\Gamma(\beta)}{\mathrm{B}(\alpha, \beta)\Gamma(t+\alpha+\beta)} = \frac{\Gamma(t+\alpha)\Gamma(\alpha+\beta)}{\Gamma(t+\alpha+\beta)\Gamma(\alpha)}. \tag{S3}$$

Observe that for $t = 0$ it holds that $d(t) = 1$. This is expected because payoffs received *now* should not be discounted. Subsequently, the average discounted payoff of player i in the repeated game with beta discounting is

$$\pi_i = \frac{\sum_{t=0}^{\infty} d(t)\pi_i(t)}{\sum_{t=0}^{\infty} d(t)}. \tag{S4}$$

Interestingly, for the case of beta discounting for $\beta > 1$ the series of the effective discount function has an explicit solution and evaluates as

$$\sum_{t=0}^{\infty} d(t) = \frac{\Gamma(\alpha)\Gamma(\beta-1)}{\mathrm{B}(\alpha,\beta)\Gamma(\alpha+\beta-1)} = 1 + \frac{\alpha}{\beta-1} = \frac{\alpha+\beta-1}{\beta-1}. \tag{S5}$$

Now, observe that after some simplification we have

$$\frac{d(t+1)}{d(t)} = \frac{\Gamma(t+1+\alpha)\Gamma(t+\alpha+\beta)}{\Gamma(t+1+\alpha+\beta)\Gamma(t+\alpha)}. \tag{S6}$$

Using the property of the Gamma function that for $z > 1$ it holds that

$$\Gamma(z+1) = z\Gamma(z),$$

we can write

$$\frac{d(t+1)}{d(t)} = \frac{(t+\alpha)\Gamma(t+\alpha)\Gamma(t+\alpha+\beta)}{(t+\alpha+\beta)\Gamma(t+\alpha+\beta)\Gamma(t+\alpha)} = \frac{t+\alpha}{t+\alpha+\beta}. \tag{S7}$$

Because $\beta > 0$, it holds that $\frac{d(t+1)}{d(t)} < 1$ and thus the effective discount function is decreasing over time, which is expected. Interestingly, as time increases the fraction $\frac{d(t+1)}{d(t)} < 1$ is *increasing*, and approaches 1 asymptotically. This implies that two distant future payoffs are discounted with a similar discount factor. Hence, also beta discounting supports the characteristic feature of hyperbolic discounting.

2 Risk-adjusted strategies in repeated games

Now, let $p_\sigma^t \in [0,1]$ denote the probability that the strategic player cooperates at the next round given that the current action profile[1] is $\sigma \in \{C,D\}^n$. By stacking the conditional probabilities for all possible outcomes into a vector, we obtain a *time dependent* memory-one strategy that determines the probability for the strategic player to cooperate in the next round:

$$\mathbf{p}^t = (p_\sigma^t)_{\sigma\in\{C,D\}^n}.$$

Accordingly, the repeated memory-one strategy given by $\mathbf{p}^{\mathrm{rep}} = (\mathbf{p}_\sigma^{\mathrm{rep}})_{\sigma\in\{C,D\}^n}$ determines the probability to cooperate when the current decision is simply repeated. Let us denote this strategic player by i. We will use the common notation $\sigma = (\sigma_i, \sigma_{-i}) \in \{C,D\}^n$. Then the entries of the repeated memory-one strategy are given by $\mathbf{p}_{(C,\sigma_{-i})}^{\mathrm{rep}} = 1$ and $\mathbf{p}_{(D,\sigma_{-i})}^{\mathrm{rep}} = 0$ for all $\sigma_{-i} \in \{C,D\}^{n-1}$. Now, let $v_\sigma(t)$ denote the probability that the outcome at round t is $\sigma \in \{C,D\}^n$, and let $v(t) = (v_\sigma(t))_{\sigma\in\{C,D\}^n}$ be the vector of outcome probabilities at round t. Using the limit of the series of the effective discounting function in (S5), the mean distribution of the action profiles is

$$\mathbf{v} = \frac{\sum_{t=0}^{\infty} d(t)v(t)}{\sum_{t=0}^{\infty} d(t)} = \frac{\beta-1}{\alpha+\beta-1}\sum_{t=0}^{\infty} d(t)v(t). \tag{S8}$$

In order to relate the average discounted payoff to the mean distribution, we introduce some additional notation adopted from[1]. Let g_σ^i denote the one-shot payoff that i receives when the action profile is $\sigma \in \{C,D\}^n$. By stacking the possible outcomes into a vector, one obtains $g^i = (g_\sigma^i)_{\sigma\in\{C,D\}^n}$, which contains all possible payoffs that player i can obtain in a round of play.

[1] The notation for the action profile $\sigma \in \{C,D\}^n$ should not be confused with the variance of the beta distribution which is indicated by σ_x^2.

The expected payoff of player i at round t can then be expressed by multiplying this one-shot payoff vector by the vector of outcome probabilities. That is, $\pi_i(t) = g^i \cdot v(t)$. Consequently, the average discounted payoff of i can be written as $\pi_i = g^i \cdot \mathbf{v}$, and thus expected payoffs of the repeated game follow from the mean distribution $\mathbf{v}$ and the one-shot payoffs. To obtain the relation between $\mathbf{p}^t$ and $\mathbf{v}$, let us define

$$u(t) := \frac{d(t+1)}{d(t)} q_C(t+1) - q_C(t) = \frac{t+\alpha}{t+\alpha+\beta} q_C(t+1) - q_C(t) = \left(\frac{t+\alpha}{t+\alpha+\beta}\mathbf{p}^t - \mathbf{p}^{\text{rep}} \right) \cdot v(t). \quad \text{(S9)}$$

Then,

$$\begin{aligned} \sum_{t=0}^{T} d(t)u(t) = d(0) &\left(\frac{\alpha}{\alpha+\beta} q_C(1) - q_C(0) \right) + \\ & d(1) \left(\frac{1+\alpha}{1+\alpha+\beta} q_C(2) - q_C(1) \right) + d(2) \left(\frac{2+\alpha}{2+\alpha+\beta} q_C(3) - q_C(2) \right) + \dots \\ & + d(T) \left(\frac{T+\alpha}{T+\alpha+\beta} q_C(T+1) - q_C(T) \right) = d(T) \frac{T+\alpha}{T+\alpha+\beta} q_C(T+1) - d(0) q_C(0). \quad \text{(S10)} \end{aligned}$$

For real $\beta > 1$ and $\alpha > 0$ we have

$$\lim_{t \to \infty} d(t) = 0. \quad \text{(S11)}$$

Thus,

$$\lim_{T\to\infty} \sum_{t=0}^{T} d(t)u(t) = \lim_{T\to\infty} \sum_{t=0}^{T} d(t) \left(\frac{t+\alpha}{t+\alpha+\beta}\mathbf{p}^t - \mathbf{p}^{\text{rep}} \right) \cdot v(t) = -q_C(0).$$

Furthermore, dividing by the series in (S5) we obtain

$$\begin{aligned} \frac{\beta-1}{\alpha+\beta-1} \sum_{t=0}^{\infty} d(t)u(t) &= \frac{\beta-1}{\alpha+\beta-1} \sum_{t=0}^{\infty} d(t) \left(\frac{t+\alpha}{t+\alpha+\beta}\mathbf{p}^t - \mathbf{p}^{\text{rep}} \right) \cdot v(t) && \text{(S12)} \\ &= \frac{\beta-1}{\alpha+\beta-1} \sum_{t=0}^{\infty} d(t)v(t) \cdot \left(\frac{t+\alpha}{t+\alpha+\beta}\mathbf{p}^t - \mathbf{p}^{\text{rep}} \right) && \text{(S13)} \\ &= -\frac{\beta-1}{\alpha+\beta-1} q_C(0) = -\frac{\beta-1}{\alpha+\beta-1} p_0, && \text{(S14)} \end{aligned}$$

where $p_0 = q_C(0)$ is the strategic player's initial probability to cooperate.

Remark 1. *The relation in* (S13) *can be seen as a probabilistic discounting extension of Akin's result on the relation between a memory-one strategy and the mean distribution of an infinitely repeated game without discounting*[2] *(Theorem 1.3).*

We are now ready to formulate a risk-adjusted strategy for repeated games with beta discounting. For this, let $\mathbb{1} = (1)_{\sigma \in \{C,D\}^n}$.

Definition 1 (risk adjusted strategy)**.** *A time dependent memory-one strategy $\mathbf{p}^t$ with entries in the closed unit interval for all time $t \geq 0$, is a risk-adjusted strategy for a symmetric n-player game if there exist shape parameters $\alpha > 0, \beta > 1$, constants $(s, l) \in \mathbb{R}^2$, weights w_j for $1 \leq j \leq n$ and ϕ such that*

$$\mathbf{p}^t = \frac{t+\alpha+\beta}{t+\alpha} \left(\mathbf{p}^{\text{rep}} + \phi \left[s g^i - \sum_{j \neq i}^{n} w_j g^j + (1-s) l \mathbb{1} \right] - \frac{\beta-1}{\alpha+\beta-1} p_0 \mathbb{1} \right), \quad \text{(S15)}$$

under the requirement that $w_i = 0$, $\sum_{j=1}^{n} w_j = 1$ and $\phi \neq 0$.

Proposition 1 (Enforcing a linear payoff relation)**.** *Assume the probabilistic discount factor has a fixed beta distribution with real parameters $\alpha > 0$ and $\beta > 1$. If a player applies a fixed risk-adjusted strategy as in Definition 1, then, independent of the fixed strategies of the $n-1$ group members, the average discounted payoffs obey the equation*

$$\pi_{-i} = s\pi_i + (1-s)l, \quad \text{(S16)}$$

Proof. Substituting the expression for $\mathbf{p}^t$ into (S13) we obtain

$$\frac{\beta-1}{\alpha+\beta-1}\sum_{t=0}^{\infty} d(t)v(t)\cdot\left(\phi\left[sg^i-\sum_{j\neq i}^{n} w_j g^j+(1-s)l\mathbb{1}\right]-\frac{\beta-1}{\alpha+\beta-1}p_0\mathbb{1}\right)=-\frac{\beta-1}{\alpha+\beta-1}p_0. \tag{S17}$$

From the distributive and commutative properties of the dot product, this implies

$$\left(\phi\left[sg^i-\sum_{j\neq i}^{n} w_j g^j+(1-s)l\mathbb{1}\right]-\frac{\beta-1}{\alpha+\beta-1}p_0\mathbb{1}\right)\cdot\mathbf{v}=-\frac{\beta-1}{\alpha+\beta-1}p_0,$$

$$\phi\left[s\pi_i-\sum_{j\neq i}^{n} w_j\pi_j+(1-s)l\right]-\frac{\beta-1}{\alpha+\beta-1}p_0=-\frac{\beta-1}{\alpha+\beta-1}p_0,$$

$$\phi\left[s\pi_i-\sum_{j\neq i}^{n} w_j\pi_j+(1-s)l\right]=0,$$

where we have used the fact that $\mathbf{v}\cdot\mathbb{1}=1$. Finally, because $\phi\neq 0$ it follows that

$$s\pi_i-\sum_{j\neq i}^{n} w_j\pi_j+(1-s)l=0 \xrightarrow{\pi_{-i}:=\sum_{j\neq i}^{n} w_j\pi_j} s\pi_i+(1-s)l=\pi_{-i}. \tag{S18}$$

□

Remark 2. *At time $t=0$, in the deterministic limit $\alpha\to\infty$ and $\beta<\infty$, the risk-adjusted strategy in* (S15) *recovers a zero-determinant strategy for infinitely repeated multiplayer games*[1]. *If $\beta=\frac{\alpha(1-\delta)}{\delta}$, then the limit $\alpha\to\infty$, recovers a zero determinant strategies for a deterministically discounted multiplayer social dilemma.*

To obtain a well-defined risk-adjusted strategy the parameters of the linear payoff relation $(s,l)\in\mathbb{R}^2$ cannot be chosen arbitrarily. This is formalized in the following definition.

Definition 2 (Enforceable linear payoff relations)**.** *A payoff relation $(s,l)\in\mathbb{R}^2$ with weights $w\in\mathbb{R}^{n-1}$ is enforceable by $\mathbf{p}^t$ if there exist shape parameters $\alpha>0,\beta>1$ such that for all $t\geq 0$ the elements of $\mathbf{p}^t$ are in the closed unit interval.*

Now, let us consider the following useful Lemma concerning the time-varying elements of the risk-adjusted strategy.

Lemma 1 (Monotonically increasing upper bounds)**.** *If the entries of $\mathbf{p}^0$ are in the closed unit interval, then the entries of $\mathbf{p}^t$ remain in the closed unit interval for all $t\geq 0$.*

Proof. It holds that

$$\begin{aligned}
&\mathbb{0}\leq\mathbf{p}^t\leq\mathbb{1},\\
&\mathbb{0}\leq\frac{t+\alpha+\beta}{t+\alpha}\left(\mathbf{p}^{\text{rep}}+\phi\left[sg^i-\sum_{j\neq i}^{n} w_j g^j+(1-s)l\mathbb{1}\right]-\frac{\beta-1}{\alpha+\beta-1}p_0\mathbb{1}\right)\leq\mathbb{1},\\
&\mathbb{0}\leq\mathbf{p}^{\text{rep}}+\phi\left[sg^i-\sum_{j\neq i}^{n} w_j g^j+(1-s)l\mathbb{1}\right]-\frac{\beta-1}{\alpha+\beta-1}p_0\mathbb{1}\leq\frac{t+\alpha}{t+\alpha+\beta}\mathbb{1}.
\end{aligned} \tag{S19}$$

Since this inequality needs to hold for all $t\geq 0$, it must hold for the minimum upper bound in t. We continue to show that this minimum occurs at $t=0$. To this end, observe that

$$\frac{\alpha}{\alpha+\beta}\leq\frac{t+\alpha}{t+\alpha+\beta}\xRightarrow{\alpha>0,\beta>0,t\geq 0} 0\leq\beta t. \tag{S20}$$

Clearly, this is satisfied for any $t\geq 0$ and $\beta>0$.

□

2.1 The existence of risk-adjusted strategies

Lemma 1 implies that the existence of risk-adjusted strategies can be shown by the implications of the inequality

$$\mathbb{0} \leq \mathbf{p}^{\text{rep}} + \phi \left[sg^i - \sum_{j \neq i}^{n} w_j g^j + (1-s)l\mathbb{1} \right] - \frac{\beta - 1}{\alpha + \beta - 1} p_0 \mathbb{1} \leq \frac{\alpha}{\alpha + \beta} \mathbb{1}, \tag{S21}$$

which leads to the following proposition.

Proposition 2. *When future payoffs are discounted by* (S3)*, generous payoff relations with* $0 < s < 1$ *and* $l = a_{n-1}$ *are not enforceable.*

Proof. Suppose all players are cooperating e.g. $\sigma = (C, C, \dots, C)$, then all players receive the one shot payoff a_{n-1}. By plugging these payoffs into the risk-adjusted strategy in Definition 1, and using the fact that $\sum_{j \neq i} w_j = 1$, one obtains

$$\mathbf{p}^t(C, C, \dots, C) = \frac{t + \alpha + \beta}{t + \alpha} \left(1 + \phi(1-s)(l - a_{n-1}) - \frac{\beta - 1}{\alpha + \beta - 1} p_0 \right). \tag{S22}$$

Using Lemma 1, the requirement that at $t = 0$ the entries of the risk-adjusted strategy are in the unit interval implies

$$0 \leq \frac{\alpha + \beta}{\alpha} \left(1 + \phi(1-s)(l - a_{n-1}) - \frac{\beta - 1}{\alpha + \beta - 1} p_0 \right) \leq 1, \tag{S23}$$

$$\frac{\beta - 1}{\alpha + \beta - 1} p_0 - 1 \leq \phi(1-s)(l - a_{n-1}) \leq \frac{\alpha}{\alpha + \beta} + \frac{\beta - 1}{\alpha + \beta - 1} p_0 - 1. \tag{S24}$$

Now for the generous strategy it is required $l = a_{n-1}$. From the above equation we obtain the requirement,

$$\frac{\beta - 1}{\alpha + \beta - 1} p_0 - 1 \leq 0 \leq \frac{\alpha}{\alpha + \beta} + \frac{\beta - 1}{\alpha + \beta - 1} p_0 - 1. \tag{S25}$$

Clearly, for any $p_0 \in [0, 1]$, the lower bound is satisfied. However, the upper bound reads as

$$0 \leq \frac{\alpha}{\alpha + \beta} + \frac{\beta - 1}{\alpha + \beta - 1} p_0 - 1. \tag{S26}$$

Because $\beta > 1$ and $\alpha > 0$, we have $\frac{\beta - 1}{\alpha + \beta - 1} > 0$ and because $p_0 \in [0, 1]$, a necessary condition for this inequality to hold for some $p_0 \in [0, 1]$ is that it holds for $p_0 = 1$, i.e.,

$$0 \leq \frac{\alpha}{\alpha + \beta} + \frac{\beta - 1}{\alpha + \beta - 1} - 1. \tag{S27}$$

We proceed to show that (S27) cannot be satisfied for $\alpha > 0$, $\beta > 1$. For this, we write (S27) equivalently as

$$0 \leq \frac{\alpha}{\alpha + \beta} + \frac{\beta - 1}{\alpha + \beta - 1} - \frac{\alpha + \beta - 1}{\alpha + \beta - 1},$$
$$0 \leq \frac{\alpha}{\alpha + \beta} - \frac{\alpha}{\alpha + \beta - 1},$$
$$\frac{\alpha}{\alpha + \beta - 1} \leq \frac{\alpha}{\alpha + \beta}.$$

Because $\beta > 1$ and $\alpha > 0$, the left hand side is positive. Moreover, because α and β are reals, they are finite and we arrive at a contradiction. This completes the proof. ☐

Remark 3 (Deterministic limits)**.** *In the deterministic limit* $\alpha \to \infty$ *and* $\beta < \infty$*, we obtain* $1 \leq 1$ *which is always satisfied and thus in the deterministic limit of infinitely repeated games without discounting, generous strategies can exist, which is consistent with the results in*[1,3]*. If we additionally let* $\beta = \frac{\alpha(1-\delta)}{\delta}$*, then*

$$\lim_{\alpha \to \infty} \frac{\alpha}{\alpha + \frac{\alpha(1-\delta)}{\delta} - 1} \leq \lim_{\alpha \to \infty} \frac{\alpha}{\alpha + \frac{\alpha(1-\delta)}{\delta}} \Rightarrow \delta \leq \delta,$$

thus generous strategies also exist in the deterministic limit of exponential discounting.

2.1.1 The generosity gap

In the previous section we have seen that under beta discounting, generous strategies are not well-defined. However, from Lemma 1 we know that over time the upper-bound of the strategies increases monotonically. This implies that at some time in the future the generous risk-adjusted strategy can become well-defined. We refer to the time before the generous strategy becomes well-defined as the generosity gap. To investigate the length of the generosity gap, one can start from (S25), but instead of evaluating it at time $t = 0$, using the general bound with a variable t. That is,

$$0 \leq \frac{\alpha + t}{\alpha + \beta + t} + \frac{\beta - 1}{\alpha + \beta - 1} p_0 - 1. \tag{S28}$$

First note that when $p_0 = 0$, this inequality cannot be satisfied for any $t \geq 0$. Therefore, assume $p_0 > 0$. After some basic manipulation one obtains that (S28) is satisfied if and only if:

$$t \geq t^* = \frac{(\alpha + \beta)(\beta - 1)(1 - p_0) + \alpha}{(\beta - 1)p_0}. \tag{S29}$$

Because $\beta > 1$ and $\alpha > 0$ this fraction is non-negative and decreasing in p_0. Thus the minimum occurs when $p_0 = 1$ and evaluates as

$$\min_{p_0 \in (0,1]} t^* = \frac{\alpha}{(\beta - 1)}. \tag{S30}$$

To obtain an understanding for how this generosity gap τ behaves with respect to increasing uncertainty we fix the mean discount factor to a constant $\mu \in (0, 1)$. That is,

$$\mu = \frac{\alpha}{\alpha + \beta} \Rightarrow \beta = \frac{\alpha(1 - \mu)}{\mu}. \tag{S31}$$

By substitution of β, the expression for the minimum generosity gap becomes

$$\tau = \frac{\alpha\mu}{\alpha(1 - \mu) - \mu}, \tag{S32}$$

that is well defined for $\alpha > \frac{\mu}{1-\mu}$, i.e. $\beta > 1$ and *decreasing* in α. By substituting the expression for β into the variance of the beta distribution we obtain

$$\sigma_x^2 = \frac{\mu^2 - \mu^3}{\alpha + \mu} < \frac{\mu(\mu - 1)^2}{2 - \mu}. \tag{S33}$$

The upper-bound on the variance is obtained by using the requirement $\alpha > \frac{\mu}{1-\mu}$. More importantly, observe that (S33) is a positive function that is *decreasing* for increasing α. For a fixed mean also the generosity gap in (S32) is decreasing for increasing α. Hence, one can conclude that the generosity gap becomes shorter as the discount rate becomes more certain. Indeed, for deterministic discount rates generous strategies exist in social dilemmas (see also Remark 3).

2.1.2 Characterizing enforceable payoff relations of risk-adjusted strategies

We now continue to characterize the enforceable payoff relations of risk-adjusted strategies. Previously, this was done for ZD strategies in infinitely repeated multiplayer social dilemmas without discounting[1]. We begin with formulating necessary conditions.

Proposition 3. *In n-player symmetric social dilemma games with payoffs as in the main text, the enforceable payoff relations of a risk-adjusted strategy with beta discount factors require the following necessary conditions*

$$\phi > 0, \tag{S34}$$

$$-\frac{1}{n-1} \leq -\min_{j \neq i} w_j < s < 1, \tag{S35}$$

$$b_0 \leq l < a_{n-1}. \tag{S36}$$

Proof. In the following, we refer to the ZD strategist as player i. Let $t = 0$ and suppose all players are cooperating e.g. $\sigma = (C, C, \dots, C)$. In this case, every player receives the one shot payoff a_{n-1}. By plugging these payoffs into the risk-adjusted strategy in Definition 1, and using the fact that $\sum_{j \neq i} w_j = 1$, one obtains

$$\mathbf{p}^0(C, C, \dots, C) = \frac{\alpha + \beta}{\alpha} \left(1 + \phi(1-s)(l - a_{n-1}) - \frac{\beta - 1}{\alpha + \beta - 1} p_0 \right). \tag{S37}$$

Now suppose on the contrary that all players defect, then all players receive the one shot payoff b_0 and the entry of the risk-adjusted strategy is

$$\mathbf{p}^0(D, D, \dots, D) = \frac{\alpha + \beta}{\alpha} \left(\phi(1-s)(l - b_0) - \frac{\beta - 1}{\alpha + \beta - 1} p_0 \right). \tag{S38}$$

The requirement that (S37) and (S38) belong to the closed unit interval results in the inequalities

$$\frac{\beta - 1}{\alpha + \beta - 1} p_0 - 1 \leq \phi(1-s)(l - a_{n-1}) \leq \frac{\alpha}{\alpha + \beta} + \frac{\beta - 1}{\alpha + \beta - 1} p_0 - 1 < 0, \tag{S39}$$

$$0 \leq \frac{\beta - 1}{\alpha + \beta - 1} p_0 \leq \phi(1-s)(l - b_0) \leq \frac{\alpha}{\alpha + \beta} + \frac{\beta - 1}{\alpha + \beta - 1} p_0, \tag{S40}$$

where the strict upper bound in (S39) follows from the fact that (S26) *cannot* be satisfied for $\alpha > 0$, $\beta > 1$ and $p_0 \in [0, 1]$. By multiplying (S39) by -1 we obtain

$$0 < 1 - \frac{\alpha}{\alpha + \beta} - \frac{\beta - 1}{\alpha + \beta - 1} p_0 \leq \phi(1-s)(a_{n-1} - l) \leq 1 - \frac{\beta - 1}{\alpha + \beta - 1} p_0. \tag{S41}$$

By adding (S40) and (S41) we obtain

$$0 < 1 - \frac{\alpha}{\alpha + \beta} \leq \phi(1-s)(a_{n-1} - b_0) \leq 1 + \frac{\alpha}{\alpha + \beta}. \tag{S42}$$

Combining this with the assumption in the main text that $a_{n-1} > b_0$, it follows that in order for the payoff relation to be enforceable it is necessary that

$$\phi(1-s) > 0. \tag{S43}$$

Now suppose there is a single defecting player, i.e., $\sigma = (C, C, \dots, D)$ or any of its permutations. In this case, the cooperators receive a_{n-2} and the single defector obtains b_{n-1}. In the case when the single defector is $j \neq i$, the entry of the risk-adjusted strategy is

$$\mathbf{p}^0_\sigma = \frac{\alpha + \beta}{\alpha} \left(1 + \phi[s a_{n-2} - (1 - w_j) a_{n-2} - w_j b_{n-1} + (1-s)l] - \frac{\beta - 1}{\alpha + \beta - 1} p_0 \right), \tag{S44}$$

and if the unique defector is i, the entry of $\mathbf{p}^{\text{rep}}$ is equal to zero and thus, the entry of the risk-adjusted strategy is

$$\mathbf{p}^0_\sigma = \frac{\alpha + \beta}{\alpha} \left(\phi[s b_{n-1} - a_{n-2} + (1-s)l] - \frac{\beta - 1}{\alpha + \beta - 1} p_0 \right). \tag{S45}$$

The requirement that (S44) and (S45) belong to the closed unit interval results in the following inequalities

$$\frac{\beta - 1}{\alpha + \beta - 1} p_0 \leq \phi[s b_{n-1} - a_{n-2} + (1-s)l] \leq \frac{\alpha}{\alpha + \beta} + \frac{\beta - 1}{\alpha + \beta - 1} p_0, \tag{S46}$$

$$\frac{\beta - 1}{\alpha + \beta - 1} p_0 - 1 \leq \phi[s a_{n-2} - (1 - w_j) a_{n-2} - w_j b_{n-1} + (1-s)l] \leq \frac{\alpha}{\alpha + \beta} + \frac{\beta - 1}{\alpha + \beta - 1} p_0 - 1. \tag{S47}$$

By combining these two conditions in a similar manner as was done for the homogeneous action profile we obtain

$$0 < 1 - \frac{\alpha}{\alpha+\beta} \leq \phi[(s+w_j)(b_{n-1} - a_{n-2})] \leq 1 + \frac{\alpha}{\alpha+\beta}. \tag{S48}$$

From the assumption $b_{z+1} > a_z$ in the main text, it follows that

$$\forall j \neq i : \quad \phi(s + w_j) > 0. \tag{S49}$$

Together with (S43) this implies that

$$\phi(1 + w_j) > 0 \xRightarrow{\exists\ j \neq i\ s.t. w_j > 0} \phi > 0.$$

This also implies that

$$\phi(1-s) \xRightarrow{\phi>0} (1-s) > 0 \Rightarrow s < 1.$$

In combination with (S49) it follows that

$$\forall j \neq i : s + w_j > 0 \Leftrightarrow \forall j \neq i : w_j > -s \Leftrightarrow \min_{j \neq i} w_j > -s. \tag{S50}$$

Moreover, because $\sum_{j=1}^{n} w_j = 1$, it follows that $\underset{j \neq i}{min}\ w_j \leq \frac{1}{n-1}$. Hence, the necessary condition turns into:

$$-\frac{1}{n-1} \leq -\min_{j \neq i} w_j < s < 1. \tag{S51}$$

Let us now investigate the bounds on the baseline payoffs. From (S40) we have

$$0 \leq \frac{\beta-1}{\alpha+\beta-1} p_0 \leq \phi(1-s)(l - b_0). \tag{S52}$$

Thus, in order for (S52) to hold it is required that

$$0 \leq \frac{\beta-1}{\alpha+\beta-1} p_0 \leq \phi(1-s)(l - b_0) \xRightarrow{\phi(1-s)>0} l \geq b_0. \tag{S53}$$

From (S39) we have

$$\phi(1-s)(l - a_{n-1}) \leq \frac{\alpha}{\alpha+\beta} + \frac{\beta-1}{\alpha+\beta-1} p_0 - 1 < 0. \tag{S54}$$

It then must hold that

$$l < a_{n-1}.$$

□

Let us now formulate the result that fully characterizes the enforceable payoff relations of risk-adjusted strategies. Towards this let $w = (w_j) \in \mathbb{R}^{n-1}$ denote the vector of weights and let $\hat{w}_z = \min_{w_h \in w} (\sum_{h=1}^{z} w_h)$ denote the sum of the j smallest weights of $j \neq i$ and finally let $\hat{w}_0 = 0$.

Proposition 4. *For the repeated n-player game with beta discount factors such that $\alpha > 0$ and $\beta > 1$ and payoffs as in the main text that satisfy the social dilemma assumptions in the main text, the payoff relation $(s, l) \in \mathbb{R}^2$ with weights $w \in \mathbb{R}^{n-1}$ is enforceable by the risk-adjusted strategy in* (S15) *if and only if $-\frac{1}{n-1} < s < 1$ and*

$$\underset{0 \leq z \leq n-1}{max} \left\{ b_z - \frac{\hat{w}_z(b_z - a_{z-1})}{(1-s)} \right\} \leq l < \underset{0 \leq z \leq n-1}{min} \left\{ a_z + \frac{\hat{w}_{n-z-1}(b_{z+1} - a_z)}{(1-s)} \right\}. \tag{S55}$$

Proof. Let $t = 0$. In the following we refer to the key player, who is employing the ZD strategy, as player i. Let $\sigma = (\sigma_1, \dots, \sigma_n)$ such that $\sigma_k \in \{C, D\}$ and let σ^C be the set of $i's$ co-players that cooperate and let σ^D be the set of $i's$ co-players that defect. Also, let $|\sigma|$ be the total number of cooperators in σ including player i. Using this notation, and using the fact that $\alpha, \beta > 0$, for some action profile σ we may write the ZD strategy in (S15) as

$$\frac{\alpha}{\alpha+\beta}\mathbf{p}^0_\sigma = \mathbf{p}^{\text{rep}} + \phi[(1-s)(l-g^i_\sigma) + \sum_{j\neq i}^n w_j(g^i_\sigma - g^j_\sigma)] - \frac{\beta-1}{\alpha+\beta-1}p_0. \tag{S56}$$

Also, note that

$$\sum_{j\neq i}^n w_j g^j_\sigma = \sum_{k\in\sigma^D} w_k g^k_\sigma + \sum_{h\in\sigma^C} w_h g^h_\sigma, \tag{S57}$$

and because $\sum_{j\neq i}^n w_j = 1$, it holds that

$$\sum_{h\in\sigma^C} w_h = 1 - \sum_{k\in\sigma^D} w_k.$$

Additionally, note that because of the symmetric one shot payoffs, for all $h \in \sigma^C$ it holds that $g^h_\sigma = a_{|\sigma|-1}$, and for all $k \in \sigma^D$, $g^k_\sigma = b_{|\sigma|}$. It follows that (S57) can be written as

$$\sum_{j\neq i}^n w_j g^j_\sigma = a_{|\sigma|-1} + \sum_{j\in\sigma^D} w_j(b_{|\sigma|} - a_{|\sigma|-1}).$$

Accordingly, the entries of the ZD strategy $\frac{\alpha}{\alpha+\beta}\mathbf{p}^0_\sigma$ are given by (S59). For all $\sigma \in \mathcal{S}$ we require that

$$0 \le \mathbf{p}^0_\sigma \le 1 \Rightarrow 0 \le \frac{\alpha}{\alpha+\beta}\mathbf{p}^0_\sigma \le \frac{\alpha}{\alpha+\beta}. \tag{S58}$$

This leads to the inequalities in (S60) and (S61). Because $\phi > 0$ can be chosen arbitrarily small, the inequalities in (S60) can be satisfied for some $\alpha > 0$ and $\beta > 1$ and $p_0 \in [0, 1]$ if and only if for all σ such that $\sigma_i = C$ the inequalities in (S62) are satisfied. Now substituting

$$\frac{\alpha}{\alpha+\beta}\mathbf{p}_\sigma = \begin{cases} 1 + \phi\left[(1-s)(l-a_{|\sigma|-1}) - \sum\limits_{j\in\sigma^D} w_j(b_{|\sigma|} - a_{|\sigma|-1})\right] - \frac{\beta-1}{\alpha+\beta-1}p_0, & \text{if } \sigma_i = C, \\ \phi\left[(1-s)(l-b_{|\sigma|}) + \sum\limits_{j\in\sigma^C} w_j(b_{|\sigma|} - a_{|\sigma|-1})\right] - \frac{\beta-1}{\alpha+\beta-1}p_0, & \text{if } \sigma_i = D, \end{cases} \tag{S59}$$

into this requirement we obtain that for all σ such that $\sigma_i = C$ the following inequalities are required to hold:

$$0 < 1 - \frac{\alpha}{\alpha+\beta} - \frac{\beta-1}{\alpha+\beta-1}p_0 \le \phi\left[(1-s)(a_{|\sigma|-1} - l) + \sum_{j\in\sigma^D} w_j(b_{|\sigma|} - a_{|\sigma|-1})\right] \le 1 - \frac{\beta-1}{\alpha+\beta-1}p_0. \tag{S60}$$

On the other hand, when $\sigma_i = D$ the following inequalities are required to hold:

$$0 \le \frac{\beta-1}{\alpha+\beta-1}p_0 \le \phi\left[(1-s)(l-b_{|\sigma|}) + \sum_{j\in\sigma^C} w_j(b_{|\sigma|} - a_{|\sigma|-1})\right] \le \frac{\alpha}{\alpha+\beta} + \frac{\beta-1}{\alpha+\beta-1}p_0. \tag{S61}$$

$$0 < (1-s)(a_{|\sigma|-1} - l) + \sum_{j\in\sigma^D} w_j(b_{|\sigma|} - a_{|\sigma|-1}). \tag{S62}$$

The inequality in (S62) together with the necessary condition that $(1-s) > 0$ implies that

$$a_{|\sigma|-1} + \frac{\sum\limits_{j\in\sigma^D} w_j(b_{|\sigma|} - a_{|\sigma|-1})}{(1-s)} > l, \tag{S63}$$

and thus provides an upper-bound on the enforceable baseline payoff l. We now turn our attention to the inequalities in (S61) that can be satisfied if and only if for all σ such that $\sigma_i = D$ the following holds

$$
\begin{aligned}
0 &\le (1-s)(l - b_{|\sigma|}) + \sum_{j\in\sigma^C} w_j (b_{|\sigma|} - a_{|\sigma|-1}) \\
&\xRightarrow{(1-s)>0} b_{|\sigma|} - \frac{\sum_{j\in\sigma^C} w_j (b_{|\sigma|} - a_{|\sigma|-1})}{(1-s)} \le l.
\end{aligned}
\tag{S64}
$$

Combining (S64) and (S63) we obtain

$$
\begin{aligned}
\max_{|\sigma| s.t. \sigma_i = D} &\left\{ b_{|\sigma|} - \frac{\sum_{l\in\sigma^C} w_l (b_{|\sigma|} - a_{|\sigma|-1})}{(1-s)} \right\} \le l, \\
l < \min_{|\sigma| s.t. \sigma_i = C} &\left\{ a_{|\sigma|-1} + \frac{\sum_{k\in\sigma^D} w_k (b_{|\sigma|} - a_{|\sigma|-1})}{(1-s)} \right\}.
\end{aligned}
\tag{S65}
$$

Because $b_{|\sigma|} - a_{|\sigma|-1} > 0$ and $(1-s) > 0$, the minima and maxima of the bounds in (S65) are achieved by choosing the w_j as small as possible. That is, the extrema of the bounds on l are achieved for those action profiles σ with $\sigma_i = C$ in which $\sum_{l\in\sigma^C} w_l$ is minimum, and those σ with $\sigma_i = D$ in which $\sum_{k\in\sigma^D} w_k$ is minimum. By the above reasoning, (S65) can be equivalently written as in the proposition. This completes the proof.

□

2.1.3 Uncertainty and the level of extortion

Define $\tilde{w}_z := \max_{w_h \in w} \sum_{h=1}^{z} w_h$ to be the maximum sum of weights for some permutation of $\sigma \in \mathcal{A}$ with z cooperating co-players. Additionally, for some given payoff relation $(s, l) \in \mathbb{R}^2$ and $w \in \mathbb{R}^{n-1}$, define

$$
\begin{aligned}
\overline{\rho}^C &:= \max_{0\le z\le n-1} (1-s)(a_z - l) + \tilde{w}_{n-z-1}(b_{z+1} - a_z), \\
\underline{\rho}^C &:= \min_{0\le z\le n-1} (1-s)(a_z - l) + \hat{w}_{n-z-1}(b_{z+1} - a_z), \\
\overline{\rho}^D &:= \max_{0\le z\le n-1} (1-s)(l - b_z) + \tilde{w}_z(b_z - a_{z-1}), \\
\underline{\rho}^D &:= \min_{0\le z\le n-1} (1-s)(l - b_z) + \hat{w}_z(b_z - a_{z-1}).
\end{aligned}
\tag{S66}
$$

Remark 4. *In the applications in the main text, we assume all weights are equal such that* $w_j = \frac{1}{n-1}$ *for all* $j \neq i$. *In this case,*

$$
\tilde{w}_z = \hat{w}_z = \frac{z}{n-1}, \quad \tilde{w}_{n-z-1} = \hat{w}_{n-z-1} = \frac{n-z-1}{n-1}.
$$

Proposition 5. *Assume* $p_0 = 0$ *and* $(s, b_0) \in \mathbb{R}^2$ *satisfy the conditions in Proposition 4, then* $\overline{\rho}^C > 0$ *and* $\overline{\rho}^D + \underline{\rho}^C > 0$. *Moreover, the threshold mean* μ *above which the extortionate payoff relation can be enforced is given by*

$$
\mu_\tau = max \left\{ \frac{\overline{\rho}^C - \underline{\rho}^C}{\overline{\rho}^C}, \frac{\overline{\rho}^D}{\overline{\rho}^D + \underline{\rho}^C} \right\}.
$$

Proof. For $\alpha > 0, \beta > 1$ and for the existence of extortionate payoff relations with $l = b_0$, we know $p_0 = 0$ is required (this fact immediately follows from the lower bound in (S40)). By substituting

this into (S60) it follows that in order for the payoff relation to be enforceable it is required that for all σ such that $\sigma_i = C$ the following holds:

$$0 < 1 - \frac{\alpha}{\alpha+\beta} \leq \phi \left[(1-s)(a_{|\sigma|-1} - l) + \sum_{j \in \sigma^D} w_j (b_{|\sigma|} - a_{|\sigma|-1}) \right] \leq 1.$$

$$\rho^C(\sigma) := (1-s)(a_{|\sigma|-1} - l) + \sum_{j \in \sigma^D} w_j (b_{|\sigma|} - a_{|\sigma|-1}) > 0. \tag{S67}$$

Hence, (S60) with $p_0 = 0$ implies that for all σ such that $\sigma_i = C$ it holds that

$$\frac{1-\mu}{\rho^C(\sigma)} \leq \phi \leq \frac{1}{\rho^C(\sigma)} \Rightarrow \frac{1-\mu}{\underline{\rho}^C(z, \hat{w}_z)} \leq \phi \leq \frac{1}{\overline{\rho}^C(z, \tilde{w}_z)}. \tag{S68}$$

Naturally, $\overline{\rho}^C \geq \underline{\rho}^C$. In the special case in which equality holds, then it follows from (S68) that $\mu \geq 0$, which is satisfied for any $\alpha, \beta > 0$. We continue to investigate the case in which $\overline{\rho}^C > \underline{\rho}^C$. In this case, a solution to (S68) for some $\phi > 0$ exists if and only if

$$\frac{1-\mu}{\underline{\rho}^C(z, \hat{w}_z)} \leq \frac{1}{\overline{\rho}^C(z, \tilde{w}_z)} \Rightarrow \mu \geq \frac{\overline{\rho}^C - \underline{\rho}^C}{\overline{\rho}^C}, \tag{S69}$$

which leads to the first expression in the proposition. Now, from (S61) with $p_0 = 0$, it follows that in order for the payoff relation to be enforceable it is necessary that

$$\forall \sigma \text{ s.t. } \sigma_i = D: \quad 0 \leq \phi \rho^D(\sigma) \leq \mu \Rightarrow 0 \leq \phi \overline{\rho}^D(z, \tilde{w}_z) \leq \mu. \tag{S70}$$

Because $\phi > 0$ is necessary for the payoff relation to be enforceable it follows that $\rho^D(\sigma) \geq 0$ for all σ such that $\sigma_i = D$. Let us first investigate the special case in which $\overline{\rho}^D(z, \tilde{w}_z) = 0$. Then (S70) is satisfied for any $\phi > 0$ and $\mu \in (0, 1)$. Now, assume $\overline{\rho}^D(z, \tilde{w}_z) > 0$. Then, (S70) and (S68) imply

$$\frac{1-\mu}{\underline{\rho}^C(z, \hat{w}_z)} \leq \phi \leq \frac{\mu}{\overline{\rho}^D(z, \tilde{w}_z)}. \tag{S71}$$

In order for such a ϕ to exist, it needs to hold that

$$\frac{1-\mu}{\underline{\rho}^C(z, \hat{w}_z)} \leq \frac{\mu}{\overline{\rho}^D(z, \tilde{w}_z)} \xrightarrow{\overline{\rho}^D,\ \underline{\rho}^C > 0} \mu \geq \frac{\overline{\rho}^D}{\overline{\rho}^D + \underline{\rho}^C}. \tag{S72}$$

This completes the proof. □

Corollary 1 (Symmetric or negatively skewed beta distribution). *Enforceable extortionate payoff relations* $(s, b_0) \in \mathbb{R}^2$ *require* $\frac{\alpha}{\alpha+\beta} \geq \frac{1}{2}$ *if one or both of the following holds:*

i) $\underline{\rho}^C \geq \overline{\rho}^D$,

ii) $\frac{1}{2}\overline{\rho}^C \geq \underline{\rho}^C$.

3 Definitions of n-player games in main text

3.1 The n-player snowdrift game

The n-player snowdrift game describes the situation in which cooperators need to clear out a snowdrift so that everyone can go on their merry way. By clearing out the snowdrift together, cooperators share a cost c required to create a fixed benefit b. If a player cooperates together with z group members, their one-shot payoff is $a_z = b - \frac{c}{z}$. If there is at least one cooperator ($z > 0$) who clears out the snowdrift, then defectors obtain a benefit $b_z = b$. If no one cooperates, the snowdrift will not be cleared and everyone's payoff is $b_0 = 0$. By applying the bounds S55 to these one-shot payoffs with $l = 0$, one can conclude that extortionate strategies can enforce any slope $s \geq 1 - \frac{c}{b(n-1)}$. Application of Proposition 5 with equal weights $w_j = \frac{1}{n-1}$ for all $j \neq i$, $b = \frac{5}{4}$, $c = 1$ and $n = 3$ leads to the left plot in Figure 4 of the main text.

3.2 The n-player linear public goods game

In the n-player linear public goods game, cooperators contribute an amount $c > 0$ to a publicly available good that grows linearly with the number of cooperators. The sum of the contributions is scaled by a public goods multiplier $1 < r < n$ and then distributed evenly among all players. For cooperators, this results in the one-shot payoffs $a_z = \frac{rc(z+1)}{n} - c$ and defectors receive $b_z = \frac{rcz}{n}$. The bounds in (S55) with $l = 0$ characterises the enforceable extortionate slopes s. An application of Proposition 5 with equal weights ($w_j = \frac{1}{n-1}$ for all $j \neq i$), the parameter values $r = \frac{12}{10}$, $c = 1$, and $n = 3$ leads to the right plot in Figure 4 of the main text.

4 References

[1] Hilbe, C., Wu, B., Traulsen, A. & Nowak, M. A. Cooperation and control in multiplayer social dilemmas. *Proceedings of the National Academy of Sciences* **111**, 16425–16430 (2014).

[2] Akin, E. The iterated prisoner's dilemma: good strategies and their dynamics. *Ergodic Theory, Advances in Dynamical Systems* 77–107 (2016).

[3] Press, W. H. & Dyson, F. J. Iterated prisoner's dilemma contains strategies that dominate any evolutionary opponent. *Proceedings of the National Academy of Sciences* **109**, 10409–10413 (2012).
